# Anti-inflammatory, antioxidant and antinociceptive activities of *Russelia coccinea* (L.) Wettst.

**Published:** 2021

**Authors:** María C. Columba-Palomares, Rosa Mariana Montiel-Ruiz, Lucia Corona Sánchez, Daniel Palafox-Gante, Verónica Rodríguez-López

**Affiliations:** 1 *Laboratory of Chemistry of Natural Products and Pharmacognosy, Faculty of Pharmacy, Autonomous University of the State of Morelos (UAEM). Morelos, México. *; 2 *Laboratory of Pharmacology, Center of Biomedical Research of the South, Mexican Institute of Social Security (IMSS). Morelos, México.*

**Keywords:** Russelia coccinea, Plantaginaceae, Anti-inflammatory activity, Antinociceptive activity, Organic extracts, Antioxidant

## Abstract

**Objective:**

Some species of the *Russelia* genus have been used different illnesses associated with pain and inflammation. The aim of this work was to characterize the biological activities (anti-inflammatory and analgesic) and antioxidant capacity of methanol and dichloromethane extracts of *Russelia coccinea*.

**Materials and Methods:**

In this study, topical anti-inflammatory activity was tested in an* in vivo* model of 12-O-tetradecanoylphorbol acetate (TPA) induced mouse ear edema of organic extracts (doses: 0.03, 0.1 and 0.3 mg/ear). The antinociceptive activity was assessed using the formalin test in mice of organic extracts (doses: 56, 100 and 300 mg/kg). Moreover, the antioxidant capacity of the extracts was determined using 2, 2-diphenyl-1-picrylhydrazyl (DPPH), 2, 2′-azinobis (3-ethylbenzothiaziline-6-sulfonate) (ABTS) and ferric reducing antioxidant power (FRAP) assays.

**Results:**

Methanol (RcM) and dichloromethane (RcD) extracts of the *R. coccinea* aerial parts were found to inhibit ear edema (48.95 and 40.13%, respectively) at a dose of 0.3 mg/ear. Acute treatment with RcM produced a significant antinociceptive effect in the late phase of formalin-induced nociception. Moreover, RcM at doses of 56, 100 and 300 mg/kg showed a significant antinociceptive effect through the early and late phases in the formalin test. RcM and RcD showed weak antioxidant capacities in the ABTS and DPPH assays; however, when their reducing capacity was evaluated by the FRAP assay, RcM showed a reducing activity similar to *Camellia sinensis* standard at the proven concentration of 1000 μg/ml.

**Conclusion:**

According to the experimental findings, the organic extracts of *R. coccinea* display remarkable antinociceptive and anti-inflammatory activities.

## Introduction

Inflammation exists in patients with infections, environmental diseases, and immune and chronic diseases (Arulselvan et al., 2016 ▶). Moreover, pain is an unpleasant sensory and emotional experience related to tissue damage, both actual or potential (Khalilzadeh et al., 2015 ▶). At present, several drugs are used for relieving pain, and in the management of inflammatory conditions, these drugs include narcotics, NSAIDs (Nonsteroidal anti-inflammatory drugs), and corticosteroids; nevertheless, these drugs may exert several adverse health effects (Ali et al., 2014 ▶).

The genus *Russelia* belonging to the family Plantaginaceae (Missouri Botanical Garden, 2019 ▶), represents species that are widely distributed across the world (Kolawole and Kolawole, 2010 ▶) but is considered native to tropical South America, especially to Mexico (Ahmed et al., 2016 ▶). Antioxidant, anti-inflammatory and antinociceptive properties have been reported for different extracts of *Russelia equisetiformis*, as well as antibacterial, antimicrobial and cytotoxic activities (Afzal et al., 2017 ▶; Ahmed et al., 2016 ▶; Awe et al., 2004 ▶; Johnson et al., 2011 ▶; Olorunju et al., 2012 ▶). Isolated compounds such as russetinol demonstrated an interesting antinociceptive effect (Awe et al., 2008 ▶) and triterpenes proved to be responsible for the anti-inflammatory activity. *Russelia sarmentosa* has been reported in ethnomedicine for its use primarily as an analgesic in Morelos, Mexico (Monroy-Ortiz and Castillo- España, 2007 ▶).

The aim of this work was to characterize the biological activities (anti-inflammatory and analgesic) and antioxidant capacity of methanol (RcM) and dichloromethane (RcD) extracts of *R. coccinea* aerial parts. To the best of our knowledge, there is no reports on biological or phytochemical activities of this species.

## Materials and Methods

**Plant material and extraction**

Aerial parts of *R. coccinea *were collected from km 3-5 of the highway Tepoztlán-Cuautla Morelos, México (March 2015). Voucher specimen No. 33907 was deposited at the Herbarium in the Centro de Investigación en Biodiversidad y Conservación (CIByC) at the Universidad Autónoma del Estado de Morelos. The air-dried and powdered parts of *R. coccinea *(265.7 g) were subjected to exhaustive extractions (5 g of dry tissue/100 ml) using n-hexane (degreased), dichloromethane (RcD) and methanol (RcM) by a maceration process at room temperature. The crude extracts were obtained following evaporation of the solvents under reduced pressure at 40°C. 

**Animals**

Groups of six male mice (CD1) weighing 25–30 g were maintained on a 12:12 hr light-dark cycle (food and water available *ad libitum*). The experiments were conducted in accordance with the federal regulations for animal experimentation care (SAGARPA, NOM-062-ZOO-1999, Mexico) and were approved by the Institutional Animal Care and Use Committee. 

**Mouse model of acute inflammation**

The mouse model of acute inflammation that was used in this study was previously reported (Columba-Palomares et al., 2018 ▶), modified by Young et al., 1983 ▶ (Young et al., 1983 ▶). Edema was induced in the right ear of each mouse by topical application of 2.5 µg of 12-O-tetradecanoyl-phorbol-13-acetate (TPA) in 20 µl of acetone. The effects of the extracts of *R. coccinea* were examined by topical application of the extracts (0.03, 0.1 and 0.3 mg/ear) to the ears (20 µl/ear, 10 µl on each surface). The mice were sacrificed 4 hr later by cervical dislocation. Ear punch biopsies (8 mm in diameter) were obtained and immediately weighed. Indomethacin (99%, Sigma‑Aldrich, USA) and TPA were dissolved in acetone. The negative control was the vehicle (acetone) with TPA (2.5 µg/ear). The percentage of inhibition was calculated according to the following expression (Rahman, 2001 ▶):

Inhibition (%) = [Edema A – Edema B/Edema A] ×100

Edema A=TPA alone (*b*-*a*);

Edema B=TPA plus sample (*b’*-*a*);

where *a* is the weight before treatment and 4 h after TPA treatment (*b*=TPA alone and *b’=*TPA plus sample).

**Evaluation of the antioxidant capacity**

**Antioxidant capacity assessed by DPPH assay**

The antioxidant activity of the extracts was determined using a previously reported method (MacDonald-Wicks et al., 2006 ▶), with minor modifications. *Camellia sinensis* (*C. sinensis*; Teavigo^®,^ epigallocatechin-3-gallate, 90%) and Trolox (Sigma, a water-soluble analog of vitamin E) were used as positive control. 

The extracts were evaluated at 156.25, 312.5, 625, 1250, 2500 and 5000 μg/ml against 175 μl of a solution of DPPH (0.025 mg/ml). After 30 min, the absorbance was measured by using a 515 nm plate reader Glomax Plate Reader (Promega). The percentage of inhibition was calculated according to the following expression:

Inhibition (%) = [A_0_ – A_1_/A_0_] ×100

Where A_0_ is the target absorbency and A_1_ is the absorbance of the extract to evaluate. The determinations were carried out in triplicate. 

**Antioxidant capacity (ABTS assay)**

The antioxidant capacity for ABTS was estimated according to that described in the literature (MacDonald-Wicks et al., 2006 ▶). The assay was performed in 96-well microplates where 20 µl of the sample (extract) was added to 230 µl of the ethanolic ABTS^•+^ solution previously adjusted to an absorbance of 0.70 (±0.1) at 754 nm. Then, 20 µl of methanol (negative control) and 20 µl of Trolox and *C. sinensis* (positive controls) were added to 230 µl of ABTS^•+^ solution, while the percentage of inhibition and the median inhibitory concentration (IC_50_) were calculated according the process described previously for the DPPH assay. 

**Ferric reducing antioxidant power (FRAP assay)**

The FRAP assay followed the method reported by Firuzi and colleagues (Firuzi et al., 2005 ▶). FRAP reagent, consisting of ferric chloride 20 mM solution of FeCl_3_•6H_2_O and 10 mM TPTZ in an acetate buffer: 300 mM (pH 3.6), in 40 mM of hydrochloric acid at a 10:1:1 proportion (v/v). The absorbances of the lower layer of samples, standards and blank at 595 nm, were measured 8 min after starting shaking. A calibration curve was prepared with a standard solution of FeSO_4_. Results are expressed as the equivalents of FeSO_4_ (mM) per gram of extract. 

**Antinociceptive assay**

**Formalin test**

The nociceptive formalin test was used as previously described (Hunskaar and Hole, 1987 ▶). Mice were placed in an open acrylic observation chamber (30 cm in diameter) for 30 min to allow them to acclimate to their surroundings. Twenty microliters of formalin (2.5%) was administered subcutaneously (s.c.) into the dorsum of the right hind paw of each mouse. Nociceptive behavior was quantified as the amount of time that the mice spent licking the injected paw for 40 min observation period. Formalin-induced licking was biphasic: the early phase (0-5 min) direct chemical stimulation of nociceptors, and the late phase (15–40 min) release in inflammatory mediators (Hunskaar and Hole, 1987 ▶; Shibata et al., 1989 ▶). The animals were orally administered with *R. coccinea* extracts (56, 100 and 300 mg/kg) 60 min before the formalin injection. Positive control (diclofenac) was administered (10 mg/kg) 15 min before the formalin injection. 

**Statistical analysis**

Data are presented as the means±SEM. Statistical evaluation was conducted using analysis of variance (ANOVA) and Bonferroni’s posttest (*p<0.05). The data were analyzed using Statgraphics Centurion XVII (Statgraphics Technologies, Inc., Virginia, USA). IC_50_ values were obtained by nonlinear regression of at least three experiments.

## Results

**Anti-inflammatory activity**

RcM significantly inhibited 48.95% of edema at a dose of 0.3 mg/ear compared to that in the negative control group (p<0.05). However, this extract exhibited a dose-dependent behavior, with 0.1 mg/ear showing an inhibition value of 24.70%. Indomethacin exhibited a significant inhibition (61.37%, p<0.05) at a dose of 0.3 mg/ear. RcD also showed activity, reaching 40.13% inhibition at the highest dose (0.3 mg/ear) (Table 1).

**Table 1:** Anti-inflammatory screening on TPA-induced mouse ear edema

**Treatments**	**Dose** **mg/ear**	**Edema Average (mg)Mean±SD**	**Edema Inhibition (%)**
RcM	0.3	13.01**±**2.36*	48.95
	0.1	19.20**±**1.96*	24.70
	0.03	--------	NA
RcD	0.3	15.26**±**1.81*	40.13
	0.1	18.15**±**0.90*	28.82
	0.03	--------	NA
Indomethacin	0.3	9.85**±**1.10*	61.37
	0.1	10.75**±**.90*	57.84
TPA		25.50**±**1.13	0.00

ANOVA, Bonferroni’s test, (n=6), *p<0.05 compared with the TPA group, RcD: Dichloromethane extract, RcM: Methanol extract, and NA: Not active at the tested concentration

**Antioxidant capacity**

RcM and RcD presented low antioxidant capacities in terms of the concentrations assayed in the DPPH assay as well as in the ABTS assay. Both extracts showed weak antiradical activity, which were less potent than the positive control. The highest ABTS cation radical scavenging activity was observed for RcD followed by RcM, with IC_50_ values of 132.89 and 219.94 μg/ml, respectively. However, these extracts showed a better reducing capacity in the FRAP test compared to *C. sinensis *(1657.72**±**32.81 µM FeSO_4_ eq), RcM (1635.80±15.01 µM FeSO_4_ eq) showed a reducing capacity similar to the positive control (Table 2).

**Table 2:** *In vitro* antioxidant capacity of *Russelia coccinea *extracts tested by DPPH, ABTS, and FRAP assays

**Treatment**	** IC**_50 _ **(µg/ml)**		**Eq (µM of FeSO**_4_ **)**
	**DPPH**	**ABTS**	**FRAP**
RcM	549.12**±**50.65	219.94**±**40.41	1635.80**±**15.01
			
RcD	962.33**±**50.65	132.89**±**24.13	723.01**±**22.33
			
*Cs	39.29**±**3.35	3.18**±**0.60	1657.72**±**32.81
			
Trolox	2.59**±**0.04	0.73**±**0.01	-----------

*C. sinensis at a concentration of 1000 μg/ml. Significantly different (p<0.05). Antioxidant activity is expressed as a greater number of equivalents of FeSO_4_.

**Antinociceptive effect**

**Figure 1:**
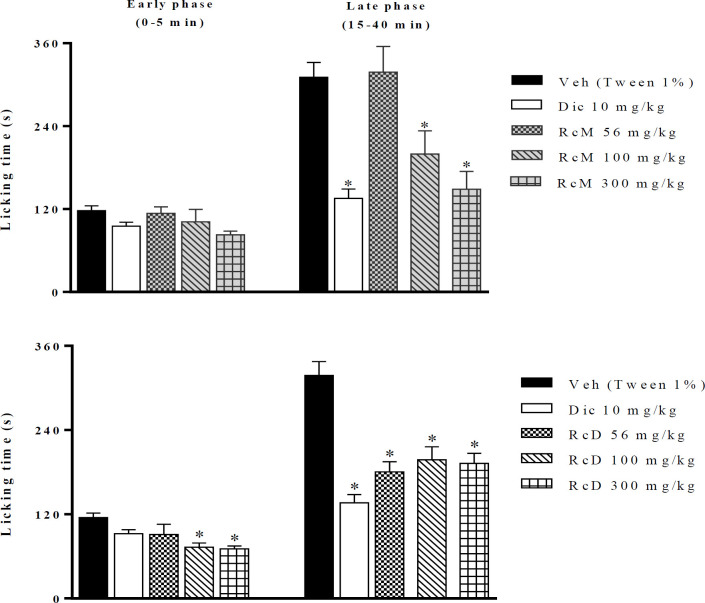
Antinociceptive effect of *Russelia coccinea* extracts. RcM: methanol extract, RcD: dichloromethane extract, and Dic: diclofenac. ***p<0.001; **p<0.01; and *p<0.05 (n=6; mean±SEM) indicate significant differences compared with the negative control group (vehicle). Significance was determined using analysis of variance (ANOVA) with a *post hoc* Bonferroni’s test

RcM at doses of 100 and 300 mg/kg (p.o.) produced a significant antinociceptive activity compared to the control group, only in the late phase (p< 0.05) (Figure 1). RcD (56, 100 and 300 mg/kg, p.o.) decreased the paw licking time in the first phase by 22, 36 and 42%, respectively, and by 41, 36 and 40%, respectively, in the second phase of the formalin test. Diclofenac suppressed the paw licking time only in the second phase of the formalin test (p< 0.05).

## Discussion

The formalin test is used to measure the behavioral effectiveness of antinociceptive agents (Hunskaar and Hole, 1987 ▶). This assay allowed local tissue injury in mouse paws and is interpreted by two distinct phases in the licking response: 1) neurogenic pain that leads to a direct chemical stimulation of nociceptors and 2) inflammatory pain triggered by a combination of stimuli, such as inflammation of the peripheral tissues and mechanisms of central sensitization (Dubuisson and Dennis, 1977 ▶; Tjølsen et al., 1992 ▶). Some evidence suggest that the second phase of the formalin test is associated with the release of several inflammatory mediators and excitatory amino acids, such as glutamate and aspartate (Khalilzadeh et al., 2015 ▶). This is the experimental model of chronic inflammation that closely resembles human arthritis, and it is possible to predict the participation of central or peripheral components in the anti-inflammatory effect of the tested compounds, although an important limitation is the very intense pain produced in the animal (Patil et al., 2019 ▶).

Inflammatory mediators (substance P and bradykinin) participate in the early phase, while histamine, serotonin, prostaglandins, nitric oxide and bradykinin, released from damaged cells, are involved in the late phase. Drugs with mainly central actions, such as narcotic analgesics, inhibit both phases, while peripherally acting drugs, such as nonsteroidal anti-inflammatory drugs and corticosteroids, inhibit only the late phase (Hunskaar and Hole, 1987 ▶; Ortiz and Castañeda-Hernández, 2008 ▶).

RcM (100 and 300 mg/kg) showed greater inhibition in the second phase than the first phase, displaying an important level of inhibition, similar to an NSAID. This suggests that the antinociceptive effect of RcM is related to its activity against inflammatory pain. Meanwhile, RcD reduced both phases of the formalin test as a partial mechanism of central action. The inhibition produced during this period could be due, at least in part, to an interaction with the opioid system. This probably indicates that the analgesic effects of the extract were mediated by inflammatory and neurogenic mechanisms. A similar effect was previously observed for the dichloromethane extract of *R. equisetiformis*, indicating central involvement of medium polarity components (Awe et al., 2008 ▶).

In the TPA model, an acute inflammatory response is observed, leading to vasodilation, platelet aggregation and leukocyte tissue infiltration. These events are the result of the activation of protein kinase C, which leads to other enzymatic processes (mitogen-activated protein kinases), therefore, an increase in arachidonic acid and its metabolites (prostaglandins, leukotrienes, etc.) and proinflammatory cytokines mediators (NF-kB, TNF-α and IL-6) (Moreno-Quirós et al., 2017 ▶; Young et al., 1983 ▶). TPA model is useful for the testing of steroidal and non-steroidal anti-inflammatory drugs, the results are usually linked to their action on cyclooxygenase (COX) and lipoxygenase (LOX). Some limitations of this model are that multiple mechanisms are involved, so it is appropriate to use this method to predict only the mode of action of anti-inflammatory compounds, instead of supporting it, sometimes animals are killed at the end of the experimental protocol to collect tissue and atrial lymph node samples for more accurate investigations (Bralley et al., 2008 ▶; Patil et al., 2019 ▶). The anti-inflammatory potential of RcM and RcD extracts observed in the murine model of TPA edema, could be due to the presence of terpene-type compounds in the extracts (Burns et al., 2000 ▶). Olonjuru and colleagues showed the presence of various triterpenoids with analgesic and anti-inflammatory effects for example, lupeol and its dose-dependent inhibition of egg albumin-induced edema and edematous response to arthritis (Olorunju et al., 2012 ▶). 

These results may suggest that the presence of pentacyclic triterpenes could be responsible for the antioxidant and anti-inflammatory effects because these metabolites have shown anti-inflammatory and antioxidative effects in several studies (Columba-Palomares et al., 2018 ▶; Romero-Estrada et al., 2016 ▶, Olorunju et al., 2012 ▶; Medeiros et al., 2007 ▶). For the above reasons, the anti-inflammatory effects observed in the extracts may be due to the presence of non-polar or medium polarity compounds. However, several studies have reported that phenolic compounds are good antioxidant and antinociceptive agents, and these compounds have been shown to inhibit COX, LOX, glutathione S-transferase, mitochondrial succinoxidase, and NADPH-oxidase, which are all enzymes involved in generating reactive oxygen species (Arulselvan et al., 2016 ▶; Johnson et al., 2011 ▶). In the literature, phenolic compounds from *R. equisetiformis* (phenylethanoid glycosides, caffeoylquinic acids, verbascoside, etc.) have been found (Afzal et al., 2017 ▶; Awe et al., 2008 ▶; Johnson et al., 2011 ▶). In the FRAP assay, RcM showed a reducing capacity similar to the positive control, so it is suggested that this effect may be associated with the presence of polar components (phenolic type) reported in the genus *Russelia*.

In conclusion, the organic extracts of *R. coccinea* showed remarkable antinociceptive and anti-inflammatory activities because they produce effects like those of nonsteroidal medications and opioids. This study is a preliminary step to identify the active compounds responsible for biological activities and their effectiveness in the treatment of various painful and inflammatory conditions.
